# Erratum: Sigurjónsdóttir, H.; Haraldsson, H. Significance of Group Composition for the Welfare of Pastured Horses. *Animals* 2019, 9, 14

**DOI:** 10.3390/ani9070453

**Published:** 2019-07-17

**Authors:** Hrefna Sigurjónsdóttir, Hans Haraldsson

**Affiliations:** 1 Faculty of Subject Teacher Education, School of Education, University of Iceland, Stakkahlíð, R105 Reykjavík, Iceland; 2 Educational Research Institute, School of Education, University of Iceland, Stakkahlíð, R105 Reykjavík, Iceland

The authors wish to make the following correction to their paper [1]:

In Table 1, citations for groups O and N were wrong.

In Table 3, there was an error in the definition of stability. The distinction between stability category 3 (one owner, minor changes) and 4 (one owner, no changes) was not made in the table, while this distinction was used in analyses.

In Supplementary Table S1, the column for stability categories contained errors. The column for proportion of males in Supplementary Table S1 contained a combination of proportions and ratios and the column for group type was missing. These errors were only present in the Supplementary Table S1 and not in the data used for analyses. 

All errors listed above have been corrected. The changes do not affect the scientific results. The authors would like to apologize for any inconvenience caused.

**Table 1:** Information on the groups.

Part^#^ of Iceland, Place, Year, Months of Study	Group ID	Females	Males *	Foals	Sub-Adults	Adults	Observation Time (hours)	Pasture Size (ha)
NW, Bessastadir—2005 (7–8)	A^37^	0	9	0	9	0	100	30
NW, Bessastadir—2005 (7–8)	B^37^	9	0	0	9	0	100	100
N, Holar—2003 (6–7)	C	17	7	0	24	0	79	5.4
W, Skaney—1997 (5–6)	D^9,35^	23	11	14	12	22	847	8
W, Skaney—1999 (5–6)	F^35^	21	10	7	6	25	488	8
SW, Litla-Thufa—2012 (7)	G	4	9	0	4	9	40	6.5
SW, Middalur—2012 (7–8)	H	6	2	1	7	1	40	30
SW, Eilifsdalur—2012 (7–8)	I	6	8	0	9	5	55	35
N, Holar—2001 (2–4)	J^33^	13	10	0	7	16	57	26.5
N, Holar—2001 (2–4)	K^33^	16	3	0	0	19	55	27.5
N, Holar—2001–2002 (12–5)	L^33^	18	10	0	20	8	102	26.5
N, Holar—2002 (1–5)	M^33^	18	12	0	0	30	81	27.5
SW, Baer—2009 (10–12)	N^39^	10	14	0	4	20	44	100
SW, Fell—2009 (1–3)	O^39^	15	23	0	6	32	41	30
* NW, Thoreyjan—2004 (7–8)	P^23^	27	1	15	0	28	76	30
* NW, Thingeyrar—2006 (6–7)	Q^23^	32	1	20	0	33	133	8
** S, Sel 1—2007 (5)	R^23,34^	18	*** 2(1)	14	3	17	81	215
** S, Sel 2—2007 (5)	S^23,34^	11	1	7	3	9	81	215
** S, Sel 3—2007 (5)	T^23,34^	29	*** 2 (1)	17	10	21	77	215
** S, Sel 4—2007 (5)	U^23,34^	26	*** 4 (3)	19	6	24	77	215

* The majority of individuals were geldings (*n =* 118), with *n =* 6 stallions and *n =* 5, 7–10-month-old colts. ** Groups with a stallion. *** Number of colts in brackets. NW is Northwest, N is North, W is West, SW is Southwest, and S is south. The superscripts on group IDs refer to previous published analyses of the data from the groups.

**Table 3:** Definitions of ranked variables which are used in the analyses.

**Age classes.** A1: 1 year, A2: 2–3 years, A3: 4–6 years, A4: 7–9 years, A5: 10–20 years, A6: 21 and more. Twenty adult horses could not be assigned to a class. In Iceland, a year is added to the age of a horse on the first day of summer in early April.
**Season.** Autumn (October–December), winter (January–April), spring (May–June), summer (July–August).
**Stability.** 0: >5 owners, 1: 4–5 owners, 2: 2–3 owners, 3: 1 owner, minor changes, 4: 1 owner, no changes. The classification assumes that the number of owners/caretakers of the horses in a group reflects the probability of changes in the composition (permanent and short-term removals and introductions). If all horses were unfamiliar in the beginning of the observation period, they got the lowest score (0).
**Stallions.** Absence in a group, (0), presence in a group, (1).
**Number of associates (friends).** The number of horses with which a horse allogrooms significantly more often than if they allogroomed with all in their group in a random manner (Chi-squared analyses, *p* < 0.05). If stay times in the groups differed between horses, the predicted values for the relevant dyads were corrected accordingly.

**Table S1:** Group characteristics.

group ID	group. size	prop. adults	no.of. foals	prop. of males	pres.of.stallions	size.of. pasture	density (horses/ha)	hay provided	aggr. median	subm. median	allogr. median	season	stability	median. no. friends	Group type
A	9	0.00	0	1.00	0	30	0.30	0	1.00	0.89	1.34	summer	0	1	Subadult group
B	9	0.00	0	0.00	0	100	0.09	0	1.20	0.95	1.69	summer	0	2	Subadult group
C	24	0.00	0	0.29	0	5.4	4.44	0	0.45	0.40	0.71	summer	1	3	Subadult group
D	34	0.51	7	0.32	0	8	4.25	1	0.06	0.08	0.26	spring	4	3	Non-breeding
F	31	0.60	14	0.32	0	8	3.88	1	0.20	0.24	0.40	spring	3	5.5	Non-breeding
G	13	0.59	0	0.69	0	6.5	2.00	0	0.29	0.33	0.81	summer	2	1	Non-breeding
H	8	0.88	1	0.25	0	30	0.27	0	0.25	0.29	0.50	summer	4	1	Non-breeding
I	14	0.56	0	0.57	0	35	0.40	0	0.23	0.20	0.71	summer	3	1	Non-breeding
J	23	0.50	0	0.43	0	26.5	0.87	1	0.21	0.33	0.39	winter	4	3	Non-breeding
K	19	1.00	0	0.16	0	27.8	0.68	1	0.51	0.62	0.62	winter	2	2	Non-breeding
L	28	0.67	0	0.36	0	26.5	1.06	1	0.55	0.69	0.20	winter	3	3	Non-breeding
M	30	1.00	0	0.40	0	27.8	1.08	1	0.59	0.78	0.19	winter	2	2	Non-breeding
N	25	0.80	0	0.58	0	100	0.25	0	0.27	0.30	0.02	autumn	3	3	Non-breeding
O	38	0.73	0	0.61	0	30	1.27	1	0.67	0.84	0.35	winter	1	2	Non-breeding
P	28	1.00	15	0.04	1	20	1.40	0	0.05	0.03	0.25	summer	1	2	Breeding group
Q	33	0.52	20	0.03	1	8	4.13	0	0.06	0.04	0.26	summer	0	1	Breeding group
R	20	0.75	14	0.10	1	200	0.10	0	0.08	0.05	0.26	spring	3	2	Breeding group
S	12	0.63	7	0.08	1	200	0.06	0	0.19	0.06	0.65	spring	4	2	Breeding group
T	31	0.57	17	0.06	1	200	0.16	0	0.04	0.04	0.32	spring	4	3	Breeding group
U	30	0.68	19	0.13	1	200	0.15	0	0.05	0.01	0.28	spring	4	2	Breeding group
