# Supplementary material for: Significance of Group Composition for the Welfare of Pastured Horses
Source: Animals (Basel). 2019 Jan 5;9(1):14. doi: 10.3390/ani9010014 (PMC6356279; doi:10.3390/ani9010014)
Supplement: Supplementary file 1 [file animals-09-00014-s001.pdf]

Table ST1. Group characteristics

group ID	group. size	prop. adults	no.of. foals	prop. of males	pres.of.stal lions	size.of. pasture	density (horses/ha)	hay provided	aggr. median	subm. median	allogr. median	season	stability	median. no. friends
A	9	0	0	1	0	30	0.3	0	1	0.89	1.34	summer	0	1
B	9	0	0	0	0	100	0.09	0	1.2	0.95	1.69	summer	0	2
C	24	0	0	0.292	0	5.4	4.444	0	0.455	0.404	0.713	summer	1	3
D	34	0.507	7	0.478	0	8	4.25	1	0.055	0.084	0.256	spring	4	3
F	31	0.605	14	0.409	0	8	3.875	1	0.204	0.242	0.396	spring	3	5.5
G	13	0.586	0	1.167	0	6.5	2	0	0.29	0.327	0.814	summer	2	1
H	8	0.875	1	0.333	0	30	0.267	0	0.255	0.291	0.5	summer	4	1
I	14	0.561	0	1.333	0	35	0.4	0	0.225	0.2	0.71	summer	3	1
J	23	0.497	0	0.769	0	26.5	0.868	1	0.211	0.335	0.387	winter	4	3
K	19	1	0	0.188	0	27.8	0.683	1	0.514	0.621	0.624	winter	3	2
L	28	0.668	0	0.556	0	26.5	1.057	1	0.551	0.694	0.205	winter	3	3
M	30	1	0	0.667	0	27.8	1.079	1	0.586	0.783	0.191	winter	4	2
N	25	0.804	0	1.5	0	100	0.25	0	0.267	0.3	0.022	autumn	2	3
O	38	0.725	0	1.533	0	30	1.267	1	0.666	0.839	0.352	winter	2	2
P	28	1	15	0.037	1	20	1.4	0	0.046	0.026	0.249	summer	4	2
Q	33	0.515	20	0.031	1	8	4.125	0	0.06	0.038	0.265	summer	4	1
R	20	0.745	14	0.176	1	200	0.1	0	0.081	0.049	0.264	spring	3	2
S	12	0.625	7	0.091	1	200	0.06	0	0.191	0.056	0.646	spring	1	2
T	31	0.574	17	0.069	1	200	0.155	0	0.039	0.039	0.324	spring	1	3
U	30	0.68	19	0.154	1	200	0.15	0	0.052	0.013	0.279	spring	0	2
